# Effect of biodegradable microneedle acupuncture for symptom relief moderate or milder atopic dermatitis: a study protocol for a multicenter, randomized, sham-controlled trial

**DOI:** 10.1186/s12906-026-05330-5

**Published:** 2026-03-09

**Authors:** Soo-Yeon Park, Min-young Jung, Cong Duc Nguyen

**Affiliations:** 1 Department of Ophthalmology, Otolaryngology & Dermatology, College of Korean Medicine, Dongshin University, 120-9, Dongsindae-gil, Naju-si, Jeollanam-do 58245 Republic of Korea; 2 College of Korean Medicine, Dongshin University, 120-9, Dongsindae-gil, Naju-si, 58245 Jeollanam-do Republic of Korea; 3 Department of Ophthalmology, Otolaryngology & Dermatology, Naju Dongshin University Korean Medicine Hospital, 14, Gyoyuk-gil, Naju-si, Jeollanam-do 58326 Republic of Korea

**Keywords:** Atopic dermatitis, Biodegradable Microneedle Acupuncture, Randomized controlled trial, Sham, Economics, Study protocol

## Abstract

**Background:**

Atopic dermatitis (AD) has no definitive cure; therefore, alternative treatments should be developed. We have demonstrated in preliminary clinical trials that Biodegradable Microneedle Acupuncture (BMA) treatment improves symptoms and is safe for mild to moderate AD.

**Aims:**

The study focused on evaluating the effectiveness and safety of a new medical device called BMA for AD, while also assessing its cost-effectiveness. It also explored the gut microbiome of patients with AD before and after BMA treatment.

**Methods:**

This multicenter, participant-assessor-blinded, sham-controlled trial will be conducted from January 10, 2025, to January 10, 2026. In total, 184 participants with AD (*n* = 92 per group) will be recruited. Participants will be assigned randomly to two equal-sized groups: the BMA and sham groups. Treatment will be administered three times per weekduring the 4-week intervention phase.

The primary outcome measure will be the objective SCORing Atopic Dermatitis index. Secondary outcome measures will include the Eczema Area and Severity Index, Dermatology Life Quality Index, Patient-Oriented Eczema Measure, and pruritus Visual Analog Scale scores.

Other outcome measures will include gut microbiome, economic and safety evaluations.

**Discussion:**

This study protocol will provide an important and thorough assessment of the effectiveness of BMA treatment in improving the symptoms of moderate or milder AD. In addition, we will evaluate the safety and costeffectiveness of BMA. We will also determine the link between AD and the microbiome.

This clinical trial has been registered with the Korean Clinical Trial Registry (registration number: KCT0009870; date of registration: 25 October 2024).

## Background

Atopic dermatitis (AD) is a chronic inflammatory dermatitis prone to flare-ups, with an increasing incidence in recent years [1]. It is a skin disease that significantly reduces the quality of life of patients and their families, but it remains difficult to treat due to the lack of a definitive cure and the side effects and high costs associated with treatment [1–3].

Currently, there is no definitive conventional cure for AD; therefore, the use of complementary and alternative therapies, such as acupuncture, is increasing [4].

Acupuncture has been shown to alleviate type 1 hypersensitivity-induced itchingin patients with AD [5] and has been reported to improve AD by blocking 5-HT receptors, thereby reducing histamine-induced acute itching [6, 7]. Acupuncture also relieves pruritus in AD by decreasing serum IL-2 and IL-4 levels while increasing interferon-γ levels in patients with chronic eczema [6]. Randomized controlled trials (RCTs) have already been conducted in AD patients to assess symptom changes following acupuncture treatment and to compare its effectiveness with sham acupuncture [8].

Biodegradable microneedle acupuncture (BMA) is an innovative medical device designed to overcome the limitations of traditional intradermal acupuncture (IDA). IDA is a form of acupuncture intended to provide prolonged stimulation by attaching a thumbtack-shaped needle to an acupointand applying continuous stimulation for a day to a week. However, long-term use of IDA can cause side effects such as foreign body sensation, oozing, and itching, particularly in individuals allergic to metal. To eliminate these adverse effects, we developed BMA using biodegradable hyaluronic acid, which has been demonstrated to be both safe and effective.

According to data from our unpublished preliminary clinical trial, we confirmedthe efficacy and safety of BMA treatment in AD. Our data indicate that BMA treatment is effective and safe in ameliorating AD symptoms.

This trial will provide a research framework to evaluate the clinical efficacy, safety, and cost-effectiveness of BMA in the treatment of patients with moderate or milder AD. We also hope that Korean medicine, including acupuncture, will play a greater rolein the management and treatment of AD.

## Methods

### Study setting

This multicenter, participant-assessor-blinded, sham-controlled trial will be conducted at two hospitals affiliated with Dongshin University: Naju Dongshin University Korean Medicine Hospital and Dongshin University Mokpo Korean Medicine Hospital, Korea. The trial has been registered with theregistered with the Clinical Research Information Service of the Republic of Korea (registration no. KCT0009870).

At the time of writing this report, enrollmenthas not started. Participant recruitment will take place from January 10, 2025, to January 10, 2026.

**Fig. 1:**
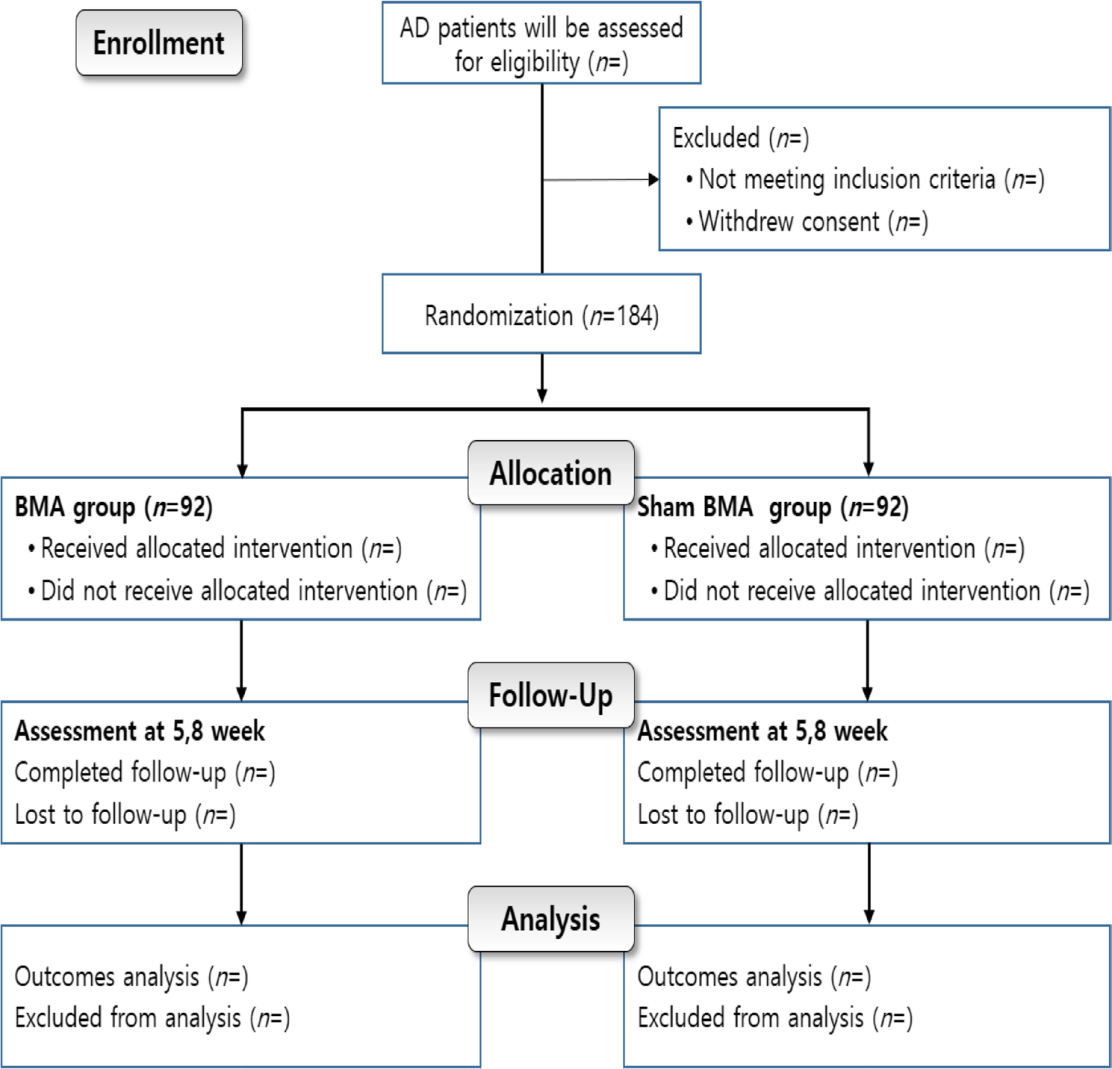
CONSORT flow diagram of the study design. This flow diagram illustrates the sequence of participant enrollment, randomization, allocation, follow-up, and analysis in the multicenter, randomized, sham-controlled trial aimed at assessing the efficacy and safety of Biodegradable Microneedle Acupuncture (BMA) for individuals with moderate or milder Atopic Dermatitis (AD). A total of 184 participants will be divided into two groups: the BMA group (*n* = 92) and the Sham BMA group (*n* = 92). Participants will be evaluated at baseline and again at weeks 5 and 8. The diagram also presents the exclusion criteria, instances of loss to follow-up, and the stages of final analysis

The present protocol satisfied the Declaration of Helsinki and was conducted following CONSORT reporting guidelines [9]. Written informed consent was obtained from each participant before registration. A flowchart of the study is shown in Fig. 1.

This flow diagram illustrates the sequence of participant enrollment, randomization, allocation, follow-up, and analysis in the multicenter, randomized, sham-controlled trial aimed at assessing the efficacy and safety of Biodegradable Microneedle Acupuncture (BMA) for individuals with moderate or milder Atopic Dermatitis (AD). A total of 184 participants will be divided into two groups: the BMA group (*n* = 92) and the Sham BMA group (*n* = 92). Participants will be evaluated at baseline and again at weeks 5 and 8. The diagram also presents the exclusion criteria, instances of loss to follow-up, and the stages of final analysis.

### Eligibility criteria

#### Inclusion criteria

Patients meeting all the following criteria will be enrolled in this study:

1. Male or female patients aged 19–70 years at the screening visit.
2. Diagnosis of AD according to the Hanifin and Rajka criteria (> 3 of 4 major symptoms and > 3 of 23 minor symptoms).
3. Patients scored 10–40 in the O-SCORAD index.
4. Patients who can read and write Korean and who voluntarily submitted written consent with their signature.

#### Exclusion criteria

Patients meeting any of the following criteria will be excluded:

1. Patients with severe skin diseases other than AD or severe oozing and maceration of the atopic lesions, who may affect the results of this study in the investigators’ opinion.
2. Patients with skin diseases due to other internal (e.g., liver diseases, renal failure), psychotic, or neurological conditions that are expected to affect the results of this study.
3. Patients who have used systemic medications, including corticosteroids, antibiotics, immunosuppressants/immunomodulators (systemic corticosteroids, cyclosporine, mycophenolate mofetil, interferon-gamma) within 1 month before the screening visit. Exceptionally, the administration of systemic antihistamines was allowed during the trial within the dose at the randomization visit if they had been administered at a constant dose for 1 month before randomization. Reduction and discontinuation were permitted, but an increase was prohibited.
4. Other severe comorbidities (e.g., heart diseases, kidney diseases, liver diseases, respiratory diseases, malignancy, immunodeficiency, etc.) deemed inadequate by the principal investigator. The abnormality criteria for liver function is defined as aspartate transaminase (AST) or alanine transaminase (ALT) level five times higher than the upper limit of the analyzed institution, and those for renal function is defined as three times higher than the upper limit of the analyzed institution.
5. Severe or uncontrolled asthma (exacerbation of asthma requiring systemic corticosteroids for at least attacks of 3 days more than two times per year)
6. Allergy to adhesives.
7. Pregnant, lactating women, or women planning pregnancy.
8. Patients deemed unsuitable by the principal investigator.

#### Dropout exclusion criteria

Each participant’s completion status will be monitored. In case of discontinuation, the reason for the discontinuation will be recorded. Cases in which the trial can be discontinued are as follows:

1. The investigators discover that the participant does not meet the inclusion or exclusion criteria.
2. The participant experiences any serious adverse events (AEs) or adverse events that make continuation in the trial challenging.
3. The participant or legal representative requests to discontinue the trial owing to unsatisfactory therapeutic effects.
4. The compliance of each participant is < 80%.
5. The investigators or participants violate the study protocol.
6. The participant withdraws consent to participate in the trial.
7. The participant is lost to follow-up.
8. The participant receives any treatment that may affect the study results without the investigator’s approval.
9. Participants deemed incapable of continuing the trial by the investigators.

### Sample size

In our previous study, the mean changes in the O-SCORAD score for 4 weeks were 9.2 ± 3.73 and 7.2 ± 3.91 in the BMA-AD and IDA-AD groups, respectively. Based on these results, we will conservatively evaluate the changes for 4 weeks in the BMA and sham devices. The number of participants required for this study was calculated as follows:

$${n}_{1}=\frac{{2({Z}_{\alpha/2}+{Z}_{\beta})}^{2}{\sigma}^{2}}{{\left(\mu1-{\mu}_{2}\right)}^{2}}=64$$

The required sample size was determined to be 64 cases per group (‘experimental group’ and ‘control group’).

$$N=\frac{{n}_{1}}{1-r}\cong92$$

Assuming a 30% drop out rate of participants at follow-up, we calculated the sample size to be 92 cases for each group and a total of 184 participants for both groups.

### Randomization and allocation

As this was a multicenter trial, stratified block randomization, with the institution as a stratification factor, will be used to randomly allocate the participants to the experimental and control groups. Randomization will be performed using the SAS 9.4 statistical software (SAS Institute Inc., Cary, NC, USA). A statistician will generate randomization codes, assign serial numbers to all 184 participants, and randomly allocate them to the two groups in a 1:1 ratio to ensure reproducibility. The codes assigned to the participants through randomization will be stored in a double-locked cabinet.

If a participant is deemed eligible after the screening procedure, the investigators will open an opaque allocation envelope containing a piece of paper labeled “experimental group” or “control group” in front of the participant. The investigators will subsequently inform the participants of the allocation results and assigned intervention. Envelopes will be stored separately.

### Blinding

Participants and outcome assessors will be blinded to the treatment group allocations. It is not possible to blind the physician; therefore, the Korean medicine doctor (KMD) who will perform the treatment will not participate in the trial outcome assessment or data collection and analysis.

Each hospital will designate several KMD trained to perform the therapy and obtain relevant operational qualifications.

The outcomes will be assessed before and after the intervention. To ensure blinding, we will choose a sham device as a placebo control in this participant-assessor-blinded clinical trial and use eye masks to shield the participants’ eyes.

The treatment device will be of the same size and material, and the treatment method will maintain a consistent frequency and intensity.

In case of serious AEs, the principal investigators at each participating center will decide whether allocation concealment needs to be broken. If allocation concealment is broken before the end of the trial, the data will be censored from the analysis and the patients will be considered to have dropped out of the trial.

### Study timeline

The study protocol comprises two main phases over 7 weeks: the screening and treatment phases (Fig. 1). The screening period will last 2 weeks, and participants will be included in this phase according to the inclusion/exclusion criteria. Participants will be treated three times a week for 4 weeks, with different interventions among the groups.

#### Screening phase 

Participants will be enrolled in the screening phase and physical examinations and inclusion evaluations will be performed. The trial’s eligible participants will be requested to complete a written informed consent form (including the procedures, risks, and options for quitting the study). The study will be thoroughly explained to the participants by a researcher with the required training. Participants will sign an informed consent form upon providing approval.

#### Treatment phase

The participants will receive interventions during the treatment phase. Participants will be checked for the O-SCORAD index, visual analog scale (VAS), Eczema Area and Severity Index (EASI), Dermatology Life Quality Index (DLQI), Patient-Oriented Eczema Measure (POEM), AEs, and compliance during the 4-week therapy period, administered three times a week.

**Fig. 2:**
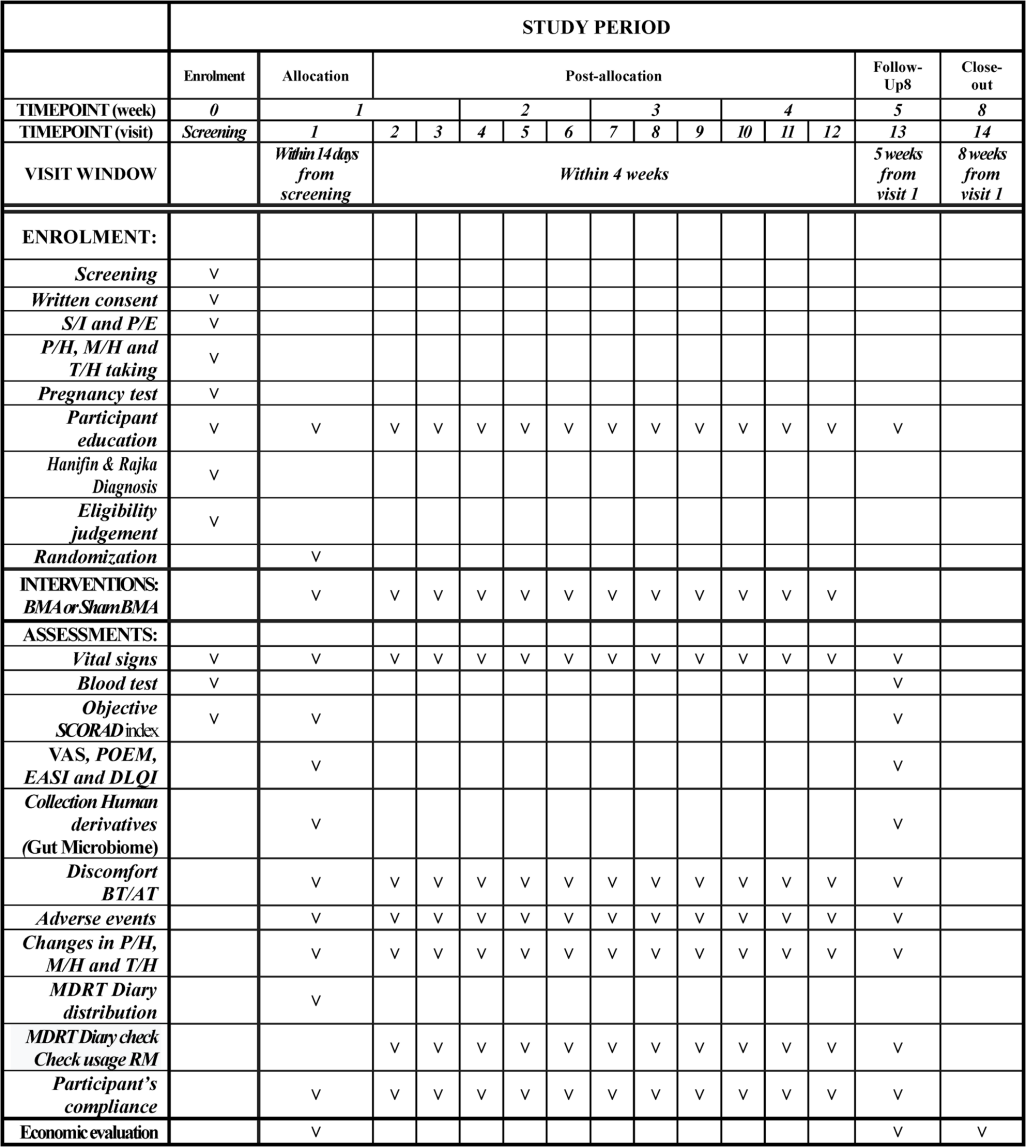
Study timeline and assessment schedule this figure presents the timeline of the study, detailing the significant phases from participant enrollment to study conclusion. It encompasses screening, randomization, intervention, and follow-up assessments over a duration of 8 weeks. Key milestones within the study include participant education, eligibility assessment, the administration of interventions (BMA or sham BMA), and clinical evaluations, all scheduled at specific intervals. The outcome assessments comprise vital signs, blood tests, SCORAD index, VAS, POEM, EASI, DLQI, collection of gut microbiome samples, monitoring of adverse events, and economic evaluations. This organized timeline facilitates the thorough monitoring of participants and the systematic collection of data throughout the trial. S/I: Sociodemographic information; P/E: Physical examination; C/P: Clinical pathology test; P/H: Past history; M/H: Medication history; T/H: Treatment history; BMA: Biodegradable microneedle acupuncture; Sham BMA: Sham Biodegradable microneedle acupuncture; O-SCORAD: Objective-SCORing Atopic Dermatitis. VAS: Visual analog scale; POEM: Patient-oriented Eczema Measure; EASI: Eczema Area and Severity Index; BT/AT discomfort: Discomfort before and after treatment; MDRT diary: Medical Device Removal Time record diary; RM : Rescue medication

After 5 weeks, all the interventions will be terminated. We will assess the demographic characteristics, medical and medication histories, skin lesion images, personal life histories, vital signs, physical examinations, and AEs (Fig. 2).

### Intervention

#### BMA / sham device treatment

**Fig. 3:**
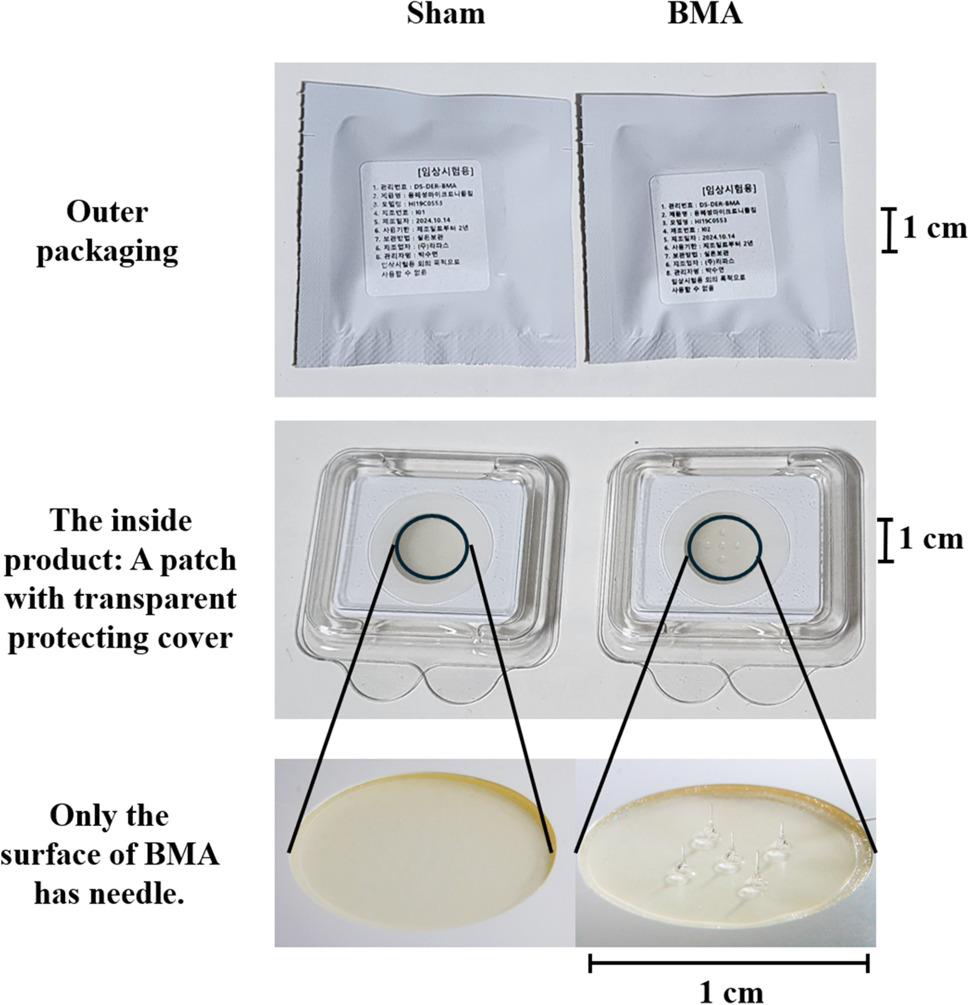
Comparison of sham and biodegradable microneedle acupuncture (BMA) Patches This figure illustrates the sham and BMA patches utilized in the research. The upper section depicts the external packaging of both patches, which are designed to be indistinguishable to maintain blinding. The central section reveals the internal product, featuring a circular patch with a clear protective layer. The lower section emphasizes a significant distinction: the sham patch is devoid of microneedles, whereas the BMA patch is equipped with biodegradable microneedles on its surface

The experimental group will receive treatment with BMA (RMD-PN1, Raphas Co. Ltd., Seoul, Republic of Korea), whereas the control group will receive sham BMA treatment (Fig. 3).

All the treatments will be administered by a KMD with more than 1 year of clinical experience in Korean medicine dermatology. The KMD will have undergone more than 10 h of training and simulation to ensure that they are able to provide identical acupuncture treatment in accordance with the predefined protocol.

**Fig. 4:**
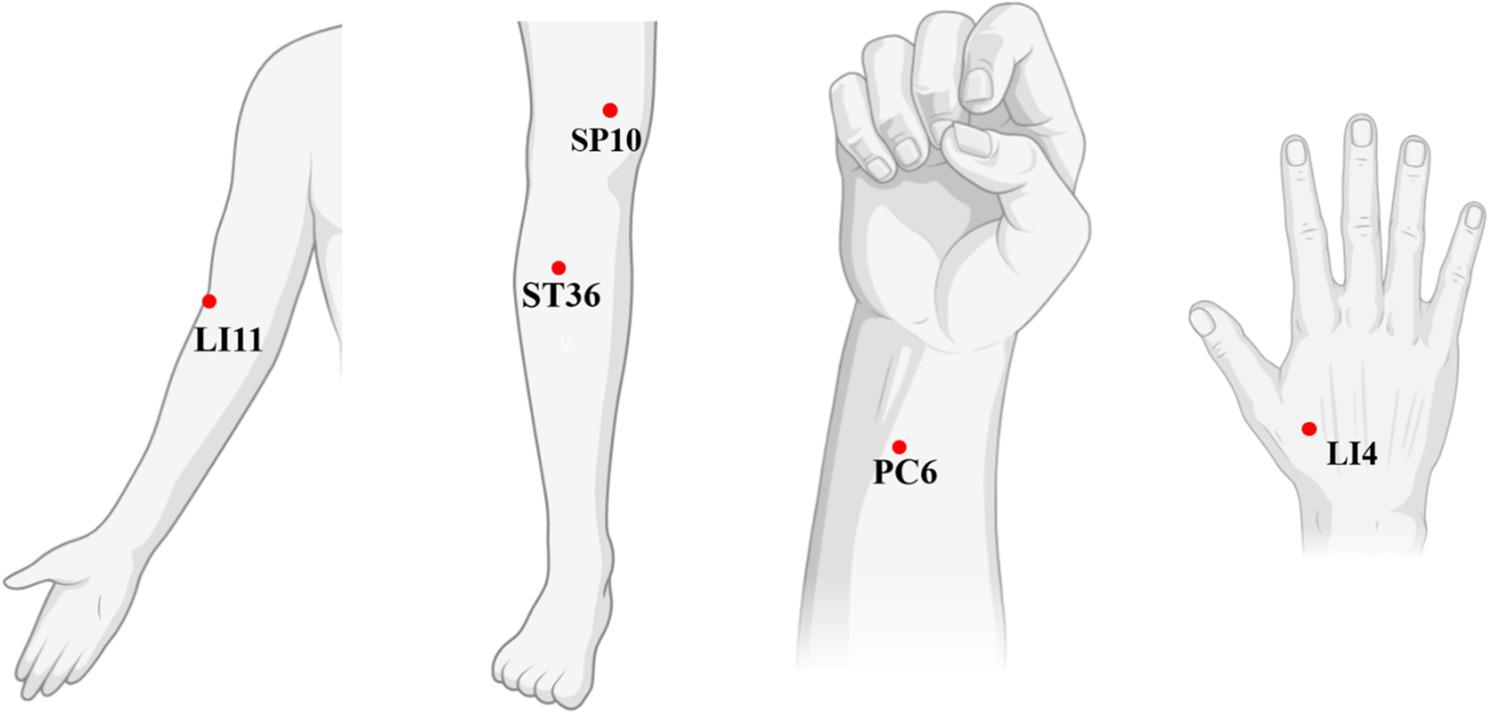
Locations of acupoints for biodegradable microneedle acupuncture (BMA) Treatment This figure depicts the five acupoints utilized in the study for BMA treatment. The chosen acupoints are LI11 (Quchi), LI4 (Hegu), PC6 (Neiguan), ST36 (Zusanli), and SP10 (Xuehai). These points are strategically positioned on the arms and legs and are frequently employed in traditional Korean medicine to manage inflammation, modulate the immune system, and treat skin-related issues. The application of BMA at these specific points is intended to amplify the therapeutic effects for symptoms associated with atopic dermatitis (AD)

A total of 184 participants with indications for the 10 acupoints (PC6, LI4, LI11, ST36, and SP10, bilaterally) (Fig. 4) will be randomly assigned to the BMA or sham device treatment groups in a 1:1 ratio. The assigned participants will be treated with BMA or a sham device.

For 4 weeks, treatments will be performed three times a week for a total of 12 times.

The investigators will instruct the participants on how to remove the BMA and disinfect the treated area themselves 4 h after treatment. The investigators will distribute Medical Device Removal Time record diaries to the participants at each visit and instruct them on the removal time record sheets.

### Participant daily life management training

Throughout the trial, all patients will receive standard basic daily life management (health guidelines) as follows.

1. Skin moisturizing and care
  i. Apply moisturizer frequently to prevent your skin from drying out. After bathing, gently pat your skin dry and apply moisturizer within 3 minutes.
  ii. Use a mild, neutral soap when bathing, and rinse out the soap thoroughly.
  iii. Avoid saunas, as they can exacerbate skin itch even more.
  iv. Bath water should be tepid, and avoid scrubbing as it can irritate the skin and exacerbate AD.
  v. Skin infections can exacerbate AD; therefore, it is essential to keep the skin and any wounds clean.
  vi. Stress can exacerbate AD by causing you to scratch your skin; therefore, it is important to relax and keep your fingernails trimmed.
2. Wear soft cotton clothing
  i. Avoid clothing made from synthetic fibers, such as coarse wool, nylon, and other non-cotton fabrics.
  ii. After washing, rinse well to remove any detergent from your clothes.
  iii. Maintain an appropriate room temperature and humidity level.
    - Maintain a temperature and humidity level that makes you feel comfortable, as sweaty conditions, such as hot rooms, heavy blankets, or clothing and high fever can exacerbate AD.
    - The optimal temperature is 22–24℃ and the optimal humidity level is 40–50%.
3. Avoid allergy triggers
  i. If you are allergic to dust mites, pollen, or animal hair, consider avoiding pets and carpets.
  ii. Avoid allergens through the air, such as formaldehyde—can cause sick building syndrome— and air pollutants.
4. Avoid irritating and allergenic foods
  i. Avoid spicy, salty, irritating, fatty, and instant foods.
  ii. Food such as milk, eggs, flour, nuts, seafood, food additives, and preservatives should be avoided.

### Rescue medication

Participants with severe pruritus may be allowed to apply Lidomex cream 0.15% (prednisolone valeroacetate) as a rescue medication in consultation with the investigators. In this case, investigators teach to apply the rescue medication with the fingertip unit method and record the use of the rescue medication at every visit.

### Combination treatment

#### Combination treatments permitted

Patients with co-morbidities or newly developed co-morbidities will be allowed to take specific medications or other treatments during the study, but only if the details are included in the medical record for analysis.

Concomitant medications and treatments that the participant has been taking prior to entering this study that the investigator believes will not affect the interpretation of the results and will be allowed at the discretion of the investigator.

#### Combination treatment prohibited

1. Systemic corticosteroids
2. Immunosuppressants
3. Any other drug or dietary supplement not recognized by the principal investigator.Concomitant treatment of any kind (including herbal treatments) related to AD is prohibited, with the exception of the rescue medication.

### Outcome measures

#### Primary outcome measure

An independent researcher will evaluate the outcomes. The schedules are listed in Fig. 2. The primary measurable outcome is the change in the O-SCORAD before (baseline) and after (5 weeks) treatment. O-SCORAD scores are designed to be measured at the screening phase, Visit 1, and Visit 13.

The O-SCORAD excludes subjective symptoms of itching and sleep disturbance from the total SCORAD [10]. A score of 15 to 40 is moderate and 1 to less than 15 is mild.

#### Secondary outcome measure

Secondary outcome measures include the EASI [11], POEM [12, 13], DLQI [14] and VAS [15].

Secondary outcome measures are designed to be measured at Visit 1, and Visit 13.

Human-derived faeces and urine will be collected to determine changes in the gut microbiome before and after treatment. These will be sent to a specializedanalytical laboratory [16].

The timeline for the efficacy evaluation is shown in Fig. 2.

#### EASI

The EASI is used to evaluate the severity of the AD symptoms. Similar to the SCORAD index, EASI is a useful evaluation tool [11]. The body is divided into four parts (head and neck, trunk, upper limb, and lower limb) to evaluate the extent of AD lesions. The severity of symptoms (erythema, edema, papules, excoriation, and lichenification) is then evaluated.

#### POEM

The POEM is a simple, easily understood, and well-validated tool for evaluating the severity of AD symptoms in the preceding week. It is known to be appropriate for use in outpatient clinic or clinical trial settings [12, 13].

#### DLQI

The DLQI is a well-validated clinical research tool that is widely used to assess the quality of life (QoL) of patients with dermatological symptoms. It evaluates the effect of skin disease on the QoL during the preceding week and consists of 10 questions [14].

#### Pruritus VAS

The pruritus VAS will be used to assess the severity of pruritus and the degree of reduction in symptoms with treatment. The VAS consists of a 100 mm scale on which participants indicate their level of pruritus by drawing a diagonal line [15].

### Safety evaluation

Several safety measures will be undertaken, including the observation of vital signs, blood test results, drug combination, physical examination, AEs, and severe AEs outcomes. The timeline for the safety evaluation is presented in Fig. 2. If an AE occurs, researchers must quickly attend to it and record the date of occurrence, severity, duration, and treatment measures. In the case of a serious AE, the researchers must take immediate action to ensure the safety of the participants, report it to the principal investigators and the Ethics Committee within 24 h, and determine if the participant should be withdrawn based on the condition.

#### Blood Tests

Blood tests will be conducted at the screening visit and visit 13 to evaluate the general health status of the participants. The examination items will include the aspartate transaminase, alanine transaminase, alanine transaminase, γ-glutamyl transpeptidase, blood urea nitrogen, and creatinine levels. Abnormal values will be reexamined at the discretion of the investigator.

### Economic evaluation

The author will also conduct an economic evaluation alongside clinical trial to evaluate the cost effectiveness of the study treatment. The utility derived from both treatment groups will be estimated in terms of Quality-Adjusted Life Years (QALYs) using EQ-5D-5 L measurement and the national tariff equation developed in South Korea [17].

### Data management and quality control

To protect the anonymity and privacy of participants, participants’ names or identifying information will remain anonymous. The data management of this trial is based on an electronic data management system, and the data administrator constructs eCRF, whose content is derived from the “case report form”. When all the subjects complete the study, all the case report forms are entered into the system. After they are reviewed by the inspectors and checked by the data administrator, the data are locked by the data administrator.

### Statistical analysis

Professional statisticians have developed a statistical analysis plan upon consultation with the primary investigators.

#### General principles

The safety and full analysis sets (FAS) of this trial will include all the participants who received any intervention at least once after randomization. The per-protocol set will only include participants in the FAS who completed the entire trial without protocol violations. The FAS is primarily used for outcome analysis, although additional outcome analyses of the per-protocol set may be performed. The safety set will be used for safety assessments, including the primary outcome of the trial. Missing values will be handled using the last observation carried-forward method.

Statistical analyses will be performed using SAS (SAS Institute Inc., Cary, NC, USA) or SPSS (IBM Corp., Armonk, NY, USA) softwares for Windows. Statistical significance is set at *p* < 0.025 for a one-tailed test and *p* < 0.05 for a two-tailed test.

#### Baseline characteristics analysis

Among the baseline characteristics of the participants, such as demographic information, continuous data will be presented as means and standard deviations, whereas categorical data will be presented as frequency tables. Continuous data will be compared using the two-sample t-test or the Mann–Whitney test, whereas categorical data will be compared using the χ^2^ test or Fisher’s exact test.

#### Primary and Secondary efficacy outcome analysis

The values of the efficacy outcomes will be presented as descriptive statistics. Differences in the values before and after the interventions will be compared using the paired t-test or Wilcoxon signed-rank test, and a 95% confidence interval will be calculated. The statistical significance of the differences between the experimental and control groups at each assessment point will be determined using a two-sample t-test or Mann–Whitney U test.

#### Safety outcomes analysis

The results of the vital signs and blood tests will be presented as means and standard deviations. Changes in the vital signs and blood test results between the initial and final visits will be compared using the paired t-test or Wilcoxon signed-rank test. The tendency towards the incidence of skin allergies will be tested using the χ^2^ test or Fisher’s exact test.

#### Economic outcomes analysis

The QALYs will be calculated using the Area Under the Curve (AUC) method. Treatment costs will be estimated by combining the frequency of each treatment and their unit costs. The economic evaluation will primarily follow the Intention-to-Treat (ITT) principle, which includes all randomized participants, regardless of adherence to the treatment protocol, to assess the effectiveness of the treatments. A secondary analysis will be performed based on the Per-Protocol (PP) principle to evaluate the sensitivity of results to missing data. For missing data in the ITT analysis, the missing data mechanism will be primarily examined using missing diagnosis method, and appropriate imputation method will be applied for building up ITT data before proceeding the analysis. The analysis period will be 8 weeks (the total follow-up period). If additional extrapolation is required, regression models or decision analysis models will be used to estimate costs and effects beyond the follow-up period. After the clinical trial period, the total analysis duration will use the 2024 South Korean currency (KRW) for cost calculations, and a 5% discount rate will be applied, in accordance with the Health Insurance Review and Assessment Service (HIRA) guidelines for cost-effectiveness evaluations. The analysis will be conducted from a societal perspective. In the base-case analysis, representative values (such as mean values) for parameters will be used, while probabilistic sensitivity analysis will be performed using all possible distributions and representative values for the estimated parameters. The results will be presented in tables, including Incremental Cost-Effectiveness Ratios (ICER), Cost-Effectiveness Planes (CEP), Cost-Effectiveness Acceptability Curves (CEAC), and Information Curve values. Statistical analysis for the economic evaluation will be conducted using Stata (MP 16.1) and R (4.3.2), and modeling for extrapolation will be performed using TreeAge Pro(Healthcare version 2023).

## Discussion

BMA was developed using biodegradable hyaluronic acid microneedle technology to mitigate the side effectsof traditional metal needle acupuncture and enhance therapeutic efficacy. Conventional IDA ismade of metal tacks for continuous needle stimulation, which can cause side effects such as foreign body sensation, skin erythema, oozing, and itching in individuals with metal allergies. To eliminate these side effects, we developed BMA using hyaluronic acid, which has been proven to be safe as a biodegradable alternative to traditional metal materials.

AD is one of a group of recurrent chronic inflammatory skin diseases. It usually begins in childhood and persists into adulthood, though it can rarely develop in middle or old age. Most individuals with AD have underlying allergic asthma, allergic rhino conjunctivitis, food allergies, and other type 1 hypersensitivity reactions [1–3, 5, 8].

The treatment of AD depends on various factors, including the clinical stage (mild, moderate, or severe), affected body surface area, patient age, comorbidities, medications, pruritus severity, and quality of life impairment. Currently, no definitive cure exists [1, 2, 8]. The goal of AD treatment is to relieve symptoms, prevent exacerbations through early intervention, reduce relapses, and ultimately control disease progression to enable patients to engage in normal social activities without discomfort [1–3, 5, 8]. Various medications, including antihistamines, corticosteroids, and immunosuppressants, are used to alleviate AD symptoms; however, due to concerns regarding side effects and resistance with long-term use, there is a need for new therapeutic approaches [4, 8].

The authors have already conducted an exploratory investigator trial to evaluate the efficacy and safety of BMA versus IDA in AD. Although the BMA group demonstrated greater improvement in AD symptoms than the control group, statistical significance was not achieved due to the small sample size (*n* = 30). Dermal allergic adverse events were observed in the IDA group, whereas no adverse events were reported in the BMA group [9].

Acupuncture has already been reported in animal and human clinical trials to alleviate itching in dermatological conditions and improve AD symptoms, making it an increasingly important complementary and alternative treatment for dermatological conditions [6, 8, 18–20].

The authors propose a prospective, multicenter, randomized controlled trial (RCT)to evaluate the efficacy, safety, and cost-effectiveness of BMA in patients with moderate or milder AD. The O-SCORAD score will be adopted as the primary outcome measure, while EASI, VAS, POEM, and DLQI scores will serve as secondary outcome measures to enhance the reliability of the results. Additionally, safety, economic evaluation, and gut microbiome analysis will be conducted.

A key limitation of this study protocol is the 5-week evaluation period, which lacks long-term follow-up.While the study can assess short-term improvement following BMA treatment, it remains unclear whether the benefits persist over time. A key strength of this study protocol is that it is the first to evaluate the efficacy, safety, and cost-effectiveness of BMA for managing atopic dermatitis symptoms.

In summary, this multicenter, randomized, sham BMA–controlled study will assess the clinical efficacy and safety of BMA in managing symptoms of moderate or milder AD.

The demand for Korean medicine (KM) therapies in the management and treatment of AD symptoms is increasing. Although the authors developed BMA, there is currently insufficient evidence to support its efficacy and safety in AD treatment. Therefore, a high-quality, multicenter, well-designed RCT is necessary to provide further evidence. This RCT aims to provide evidence that BMA safely improves AD symptoms in patients with sensitive skin while simultaneously assessing its economic feasibility to support broader coverage.

## Data Availability

The data that support the findings of this study are available on request from the corresponding author. The data are not publicly available due to privacy or ethical restrictions.

## Abbreviations

AD: Atopic dermatitis
BMA: Biodegradable microneedle acupuncture
IDA: Intradermal acupuncture
O-SCORAD: Objective-SCORing Atopic Dermatitis
AST: Aspartate transaminase
ALT: Alanine transaminase
KMD: Korean medicine doctor
VAS: Visual analog scale
EASI: Eczema Area and Severity Index
DLQI: Dermatology Life Quality Index
POEM: Patient-Oriented Eczema Measure
eCRF: Electronic Case Report Form
AE: Adverse event
FAS: Full analysis set
QoL: Quality of life
KM: Korean medicine
